# Reproductive and Behavioral Evaluation of a New Immunocastration Dog Vaccine

**DOI:** 10.3390/ani10020226

**Published:** 2020-01-31

**Authors:** Daniela Siel, María José Ubilla, Sonia Vidal, Alexandra Loaiza, John Quiroga, Federico Cifuentes, Timothy Hardman, Lisette Lapierre, Rodolfo Paredes, Leonardo Sáenz

**Affiliations:** 1 Laboratory of Veterinary Vaccines, Department of Animal Biology, Faculty of Veterinary and Animal Science, Universidad de Chile, Santiago 8820808, Chile; danisiel@uchile.cl (D.S.); svidalvilches@gmail.com (S.V.); alexa.loaiza.farias@gmail.com (A.L.); john.quiroga@uach.cl (J.Q.); thardman@students.pitzer.edu (T.H.); 2 Escuela de Medicina Veterinaria, Facultad de Ciencias de la Vida, Universidad Andres Bello, Santiago 8370146, Chile; maria.ubilla@unab.cl (M.J.U.); rparedes@unab.cl (R.P.); 3 Department of Animal Pathology, Faculty of Veterinary and Animal Sciences, Universidad de Chile, Santiago 8820808, Chile; federico.cifuentes@uchile.cl; 4 Department of Preventive Medicine, Faculty of Veterinary and Animal Science, Universidad de Chile, Santiago 8820808, Chile; llapierre@uchile.cl

**Keywords:** GnRH, immunocastration, vaccine, dog, non-surgical sterilization, reproduction

## Abstract

**Simple Summary:**

Population control of free-roaming dogs is a topic of great interest worldwide. Immunocastration (immune blockade of GnRH-I, the hormone that commands reproductive ability) has emerged as a complementary alternative to surgical castration. In this work, the effectiveness of an immunocastration vaccine for dogs was evaluated, as were the reproductive and behavioral characteristics of vaccinated animals. Two consecutive trials were carried out for this purpose. A first trial was conducted under experimental conditions, observing an immune response against the vaccine over a nine-month period that was associated with changes in the testicular function of the animals (decrease in testosterone and alteration of the characteristics of the ejaculate). The second trial was conducted on dogs who had owners, and vaccinated animals showed an immune response against the five-month vaccine and a decrease in unwanted behaviors associated with the presence of sex hormones. While more studies are needed, this vaccine is emerging as a promising tool for the reproductive and behavioral management of male dogs.

**Abstract:**

Canine immunocastration development has been of interest for many years as a complementary strategy to surgical castration. The purpose of this paper was to verify the effect of a recombinant vaccine for dog immunocastration. Two tests were done, one under controlled conditions and a second under field conditions. Animals were injected with 1 mL of 500 µg GnRXG/Q recombinant protein; 500 µg of low molecular weight chitosan as adjuvant; 1 mL NaCl 0.9% q.s. In the first trial, eight Beagle male dogs between the ages of 1 and 3 comprised the sample, randomly divided into two groups: vaccinated group (*n* = 7) and control group (*n* = 2). The second trial had 32 dogs with owners. In the first controlled conditions trial, the vaccine produced specific antibodies that remained until the end of the trial (day 270), inducing reduced testosterone and spermiogram changes in the immunized animals. In a second trial, on the field, specific immunity was induced, which remained high up to day 150. The vaccine also reduced sexual agonistic and marking behaviors. This new vaccine proved to be safe, immunogenic, capable of reducing gonadal functionality, and had a positive effect on inducing reduced sexual, agonistic, and marking behavior of the animals.

## 1. Introduction

Dogs play an important role in our society, mainly as pets. However, because of lack of responsible ownership by pet owners—among other things—the abandoned and free-roaming dog population has increased, posing a high risk to public, environment, and animal health [1,2,3,4]. In Chile, a steady increase has been seen in the free-roaming dogs, with and without owners [5,6,7]. This situation makes it imperative to find strategies that maintain animal wellbeing as well as protecting human and animal health. Wellbeing is altered in free-roaming dogs due to high mortality, malnutrition, starvation, disease, and abuse, among others [4]. 

Human beings have worked for years to control dogs’ sexual behaviors which are undesirable for humans, such as marking, aggressive behavior, and unwanted crosses. To this end, spaying (females), and neutering (males) has been the most utilized method [8]. However, the interest in developing non-surgical methods has increased due to the fact that in many countries, especially developing countries, the large number of animals makes surgical methods almost impossible. This is mainly due to high implementation costs, a need for qualified professionals to perform surgeries and anesthesia, as well as operating rooms and equipment and medical care after surgery [8,9]. Hence, cheaper, easily applied non-surgical methods based on hormones antibodies, and other pharmacological techniques have been researched and developed to be used on both genders; among these the most promising method is immunocastration [8,10]. 

Immunocastration is a vaccination against the gonadotropin-releasing hormone (GnRH-I). The GnRH-I is a hormone produced and secreted in the hypothalamus, reaching the receptors in the adenohypophysis via the hypothalamic-pituitary portal [11]. Bonding of GnRH-I to its receptors stimulates pituitary secretion of the follicle-stimulating hormone (FSH) and the luteinizing hormone (LH), which are the key reproductive hormones [8]. Immunocastration creates specific antibodies that recognize and bond to the hormone GnRH-I, preventing bonding with pituitary receptors, thus inhibiting FSH and LH secretion, causing infertility [10,12]. Thus, immunocastration has proven inhibition of reproductive activity in pigs [13,14,15], cattle [16,17], horses [18], sheep [19], mice [20,21,22,23], camelids [24], cats [25], chickens [26], and dogs [9,27,28]. Bansal and collaborators (2019) have recently developed and proven that immunogenicity of a vaccine able to develop immunity against GnRH and rabies virus simultaneously in mice [29].

Most dog-related studies have evaluated the immunological and reproductive effects of immunocastration [9,27,28], but have not identified the effects on sexual and social behavior in dogs with owners, in field conditions.

The purpose of this paper was to evaluate the effect of a new immunocastration recombinant vaccine on the immunological, reproductive, and behavior parameters in domestic canines. A trial under controlled conditions was done first (270 days), and subsequently, a field conditions trial was done on dogs with owners (180 days). Castrations and histological determinations of the gonads were performed one month after (day 210) the end of the controlled trial. The vaccine used in both trials had previously inhibited rats’ reproductive function [21,22], and other species such as pigs and cattle (unpublished data).

## 2. Materials and Methods

### 2.1. Vaccine Formula

#### 2.1.1. Antigen

The GnRXG/Q antigen is a recombinant protein that includes the amino acidic sequence of the GnRH-I hormone, flanked by a spacing sequence to improve the immunogenicity of GnRH. This antigen has been successfully used before in our laboratory, proving an immunocastrating effect in rats [20,21,22]. 

#### 2.1.2. Adjuvant

A suitable adjuvant is required in the vaccine formula [21,30] because it is a recombinant antigen and GnRH-I is immunogenically low as it is a small self-peptide (ten amino acids). Low molecular weight chitosan (Sigma Aldrich) was used as an adjuvant in this trial; it has proven to be effective as a carrier and immune potentiator in previous rat trials [21,22].

### 2.2. Controlled Conditions Trials

#### 2.2.1. Animals and Housing

Fourteen male Beagles, between the ages of 1 and 3 years old were randomly divided into two groups: vaccinated (*n* = 7) and the control group (*n* = 7). The dogs were housed individually under ongoing confinement in 3.2 m^2^ kennels with a 4.0 m^2^ exercise area. The animals received balls and food puzzles as environmental enrichment, and there was daily contact with humans that included playtime and two daily walks (morning and afternoon).

The temperature in the kennels was between 18 and 24 °C, and they were cleaned twice a day to keep the air free of ammonia gas and purin smell. Light/darkness hour ratio was 12/12. The diet was based on commercial extruded feed, two daily portions and unrestricted access to potable drinking water. 

Only healthy dogs participated, corroborated before by a clinical examination, biochemical profile, hemogram, and brucellosis diagnosis. Vaccines and deworming were done according to recommendations related to the species.

After the study, animals were kept in the Veterinary Faculty at the Universidad de Chile, in order to be used for other research projects at the University. Every animal handling protocol was approved before by the Universidad de Santiago de Chile bio ethical committee (certificate No.132016).

#### 2.2.2. Trial Duration

The effectiveness trial under controlled conditions lasted 270 days. The animals acclimatized during 28 days before the trial.

#### 2.2.3. Vaccination and Blood Collection

The animals were injected with 1mL of 500 µg GnRXG/Q recombinant protein; 500 µg of low molecular weight chitosan as adjuvant; 1 mL NaCl 0.9% q.s. [21,22]. Two vaccinations were done, on day 1 and 30 of the trial; subcutaneous in the interscapular area, with 5 mL syringes and 21 G needles. The area was disinfected with 70% (v/v) ethyl alcohol-soaked cotton wool.

To extract the animals’ serum blood samples were taken every month from the cephalic vein, from day 1 to 270 of the trials.

#### 2.2.4. Vaccine Safety

The dogs were monitored daily after each immunization to check for local and/or systemic symptoms. The observation period was extended from the first vaccine up to 10 days after the second dose. Using the established score, palpation of the injection spot was done, and the local and/or systemic reaction was evaluated.

To discard unwanted systemic effects a hemogram and a biochemical profile were done—as mentioned before—at the beginning and the end of the trial.

#### 2.2.5. Establishing Immunoglobulin Production

The immunogenic capacity estimate of the vaccine was done measuring the specific antibodies (IgG) against the recombinant GnRX G/Q protein with an indirect enzyme-linked immunosorbent (ELISA) assay, using the protocol described before [21,22] but with some changes, using a serum dilution of 1:250 and a rabbit’s secondary polyclonal antibody, anti-canine IgG conjugated with peroxidase (Jackson Immunoresearch Laboratories) in a 1:5000 dilution in a diluting buffer.

#### 2.2.6. Serum Testosterone Evaluation

An ELISA assay was done to evaluate testosterone levels, with the testosterone ELISA assay kit (DRG Instruments GmbH, Germany, Division of DRG International, Inc.), as per manufacturer’s instructions. A 96-well plate coated with anti-Testosterone antibodies and preblocked were incubated with 25 µL of each standard (kit), control (kit), and serum from the trial animals (dilution 1:10, with Standard 0, kit) and 200 µL enzyme conjugate (kit) during 1 h at ambient temperature. After incubation, the plates were washed three times with a washing solution (kit) and incubated during 15 min with a 200 µL substrate solution (kit). The reaction was terminated with 100 µL Stop Solution (kit) and absorbency was immediately read at 450 nm.

Blood samples for these evaluations were obtained from all animals at the same time (between 8:00 and 11:00) on days 1, 30, 90, 180, and 270 for the controlled assay and on days 1, 30, 60, 90, 120, 150, and 180 for the field assay.

#### 2.2.7. Spermiogram Evaluation

To complement the results associated with the testosterone serum levels, semen was extracted at the end of the trial (the three spermatic fractions) from all the injected and control animals by manual stimulation to then evaluate ejaculate characteristics, taking into consideration the reference values for the species.

### 2.3. Trial under Field Conditions

After the controlled conditions trial was finished, 32 dogs of different breeds and sizes, between 1 and 9 years old, with owners residing in Santiago or Valparaíso, Chile, were vaccinated with the same formulation used in the previous trial. Housing, feeding, and upkeep of the animals was the responsibility of each animal’s owner, under ongoing oversight through visits and telephone calls. Each owner signed an informed consent form before starting the trial.

Vaccinations, blood extraction, and immunoglobulins and testosterone determination were done as mentioned in Section 2.2.3, Section 2.2.5 and Section 2.2.6. The test lasted 180 days.

Owners were offered surgical castration—at the end of the trial—as definitive sterilization of their dogs.

The safety of the vaccine was confirmed with a biochemical profile and hemogram at the beginning and end of the trial; with ongoing communication with the owners, receiving information related to the appearance of the injection spot and the overall health of the animal during the trial.

Only healthy dogs participated, corroborated before by a clinical examination, biochemical profile, hemogram, and brucellosis diagnosis. Vaccines and deworming were done according to recommendations related to the species. 

Every animal handling protocol was approved before by the Universidad de Santiago de Chile bio ethical committee (certificate No.132016).

#### 2.3.1. Histological Gonadal Changes Evaluation

On day 210, a month after the trial ended, 25 dogs were surgically castrated (based on the owner’s approval) using a routine surgical technique and inhalation anesthesia. Once removed, the testicles were treated with a buffered formaldehyde solution at 10% (v/v), serially cut to 5 μm thickness, placed on an object holder, and dyed with hematoxylin and eosin stain.

The specimens were classified as (i) normal, seminiferous tubules of homogeneous diameter, with the germinal seminiferous epithelial layers preserved. Testicular interstice with normal characteristics; (ii) atrophic testicle, loss of the seminiferous epithelium, tubular remains, and fibrosis observed in the testicular parenchyma.

#### 2.3.2. Behavioral Evaluation

**Figure 1:**
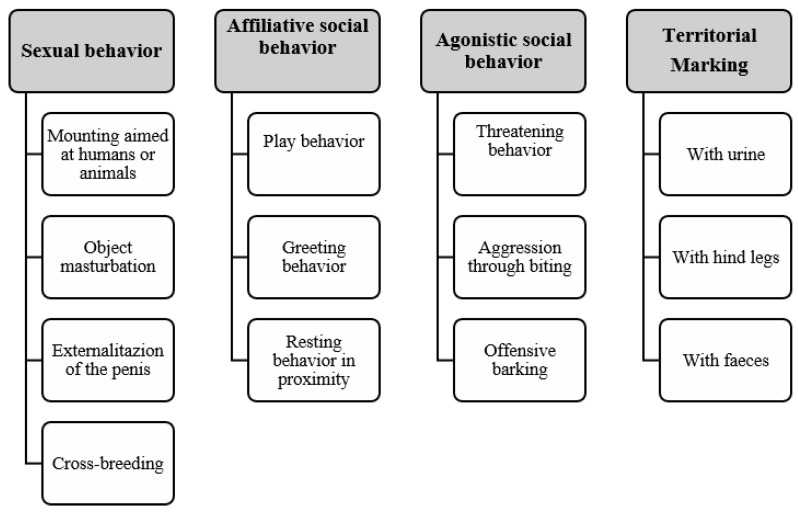
Behavioral groups and behavior subcategories considered in the survey directed at dog owners in the study.

Four evaluations were done (days 1, 60, 120, and 180) with visits with an owner questionnaire, showing explicit supporting images representing each behavior. The questionnaire was divided into four behavioral categories, each with different subcategories (Figure 1). The surveyed owners responded based on three frequency possibilities: never, sometimes, or always.

### 2.4. Statistical Analysis

In the controlled trial, the IgG and testosterone levels were analyzed with the two-factor Kruskal Wallis test and after the Bonferroni multiple comparisons test was done.

The results related to the IgG and testosterone in the field trial were done with the Paired Sample *t*-Test. The behavior evaluation was done with the Kruskal Wallis test and after the Tukey multiple comparisons test was executed.

The GraphPad Prism© version 5.01 software was used for the analyses. The differences were regarded as significant with values of *p* < 0.05.

## 3. Results

### 3.1. Safety

No local or systemic adverse effects because of the vaccination were observed in both trials. In the controlled conditions trial, two of the injected animals showed a slight reaction (palpable but not visible) up to four days post-vaccination becoming unnoticeable later; except for one that showed a palpable lesion up to the sixth day after the first vaccine, fully disappearing afterward. In the field trials, none of the participating animals’ owners noticed a local reaction after the vaccination.

### 3.2. Controlled Conditions Trial

#### 3.2.1. IgG Immunoglobulin and Serum Testosterone Production

**Figure 2:**
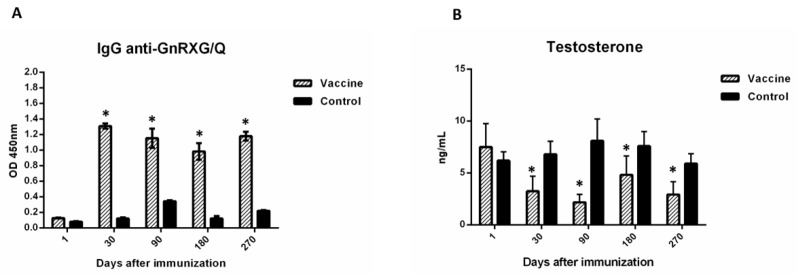
Vaccination induced the production of the specific IgG anti GnRXG/Q and decrease serum testosterone in controlled conditions. Dogs were inoculated on days 1 and 30 with the GnRXG/Q antigen and chitosan as adjuvant (Vaccine group, *n* = 7) or chitosan in absence of the antigen (Control group, *n* = 7). Vaccination induced the production of specific IgG anti GnRXG/Q (**A**) and a decrease in serum testosterone (**B**) from the first vaccination (day 30) to the end of the study. All data are represented as mean ± SEM. Asterisks (*) indicate significant differences in relation to day 0 (*p* < 0.01).

Figure 2 shows that when the IgG serum production is compared against the GnRXG/Q recombinant antigen, that from the first vaccination and until the end of the trial (day 270), the immunized animals displayed a significant increase of IgG compared to the control group (Figure 2A).

The evaluation of the testosterone serum levels revealed that from the first vaccination and until the end of the trial, the injected animals had a lower testosterone level than the control group (Figure 2B).

#### 3.2.2. Spermiogram Evaluation

Table 1 shows that there was at least one change in the ejaculate in six of the seven vaccinates animals. Five of the seven vaccinated dogs showed a lower than normal sperm concentration, together with a normal sperm morphology change in dogs 2 and 6. The progressive motility of the sperms in dogs 1 and 6 changed. Dog 7 did not have any sperm in all the ejaculate fractions, therefore, all the parameters had zero value.

In the control group, semen extraction could only be carried out on two of the seven dogs, given that it was not possible to induce ejaculation in the other five dogs. The two dogs evaluated in the control group did not show any changes in the evaluated parameters (Table 1).

### 3.3. Field Trial 

#### 3.3.1. IgG Immunoglobulin and Serum Testosterone Production 

**Figure 3:**
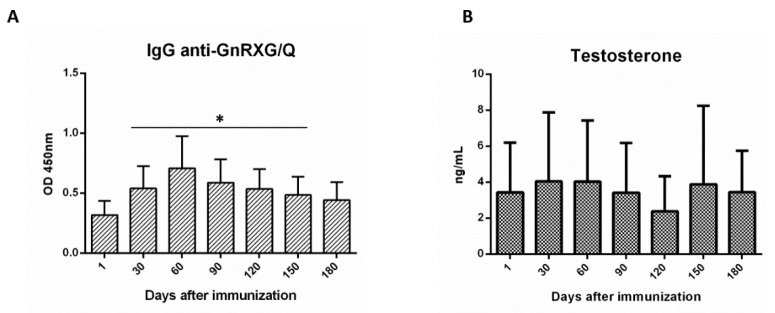
Vaccination induced the production of the specific IgG anti GnRXG/Q but did not decrease serum testosterone in field conditions. Dogs (*n* = 32) were immunized on days 1 and 30 with GnRXG/Q antigen and chitosan as adjuvant. Vaccination induces specific IgG anti GnRXG/Q from day 30 to day 150 of the study (**A**). No significant differences in testosterone production were observed throughout the study (**B**). Production of serum IgG against the GnRxG/Q antigen was determined by indirect ELISA and serum testosterone by chemiluminescence. All data are represented as mean ± SEM. Asterisks (*) indicate significant differences in relation to day 0 (*p* < 0.05).

Figure 3 shows an increase of IgG serum in the injected animals related to the beginning of the study and that the levels remain until day 150 of the trial, dropping on day 180 (Figure 3A). Therefore, on day 180 the test was concluded, in order to avoid possible animal reproduction, given that they were not sterilized by the immunocastration. 

There were no differences in testosterone serum throughout the trial, probably because of the high variability evidenced between the participants (Figure 3B). No significant differences were found (data not shown) in the same participant between dates.

#### 3.3.2. Histological Gonadal Changes

**Figure 4:**
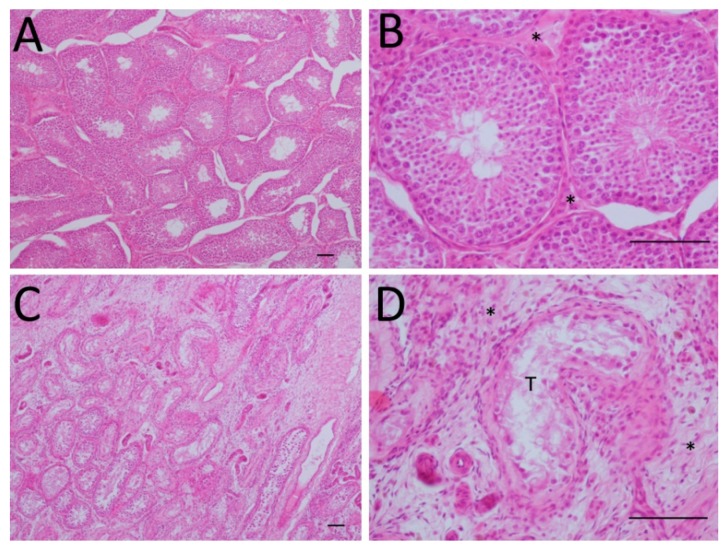
Histological alterations in the testicular parenchyma due to vaccination. One month after the end of the study (day 210), 25 animals were surgically castrated for testicular parenchyma evaluation. Of these, 20 dogs presented “normal” testicular parenchyma with seminiferous tubules with normal structure and spermatocyte maturation (**A**) and an interstitium with few cells and scarce amount of connective tissue (asterisks) (**B**). Five dogs presented testicular degeneration or atrophy characterized by remaining seminiferous tubules having an irregular profile and separated by connective tissue (**C**) and seminiferous tubules with loss of most maturation layers and thickened basement membrane with an irregular profile. Interstitium is expanded by fibrosis and few inflammatory cells (asterisks) (**D**). Five-micrometer slices were stained with hematoxylin–eosin to analyze testicular parenchyma. A and C: Bar 200μm; B and D: Bar 100 μm.

One month after the trial ended (day 210), five of the 25 testicles analyzed were atrophic and 20 showed normal histological characteristics (Figure 4). The animals with “normal” histology displayed a structure preserved in the tubules, without alteration in the seminiferous epithelium (Figure 4A), with normal interstitial tissue, and few connective tissue (Figure 4B). The animals with “atrophic testicles” showed a loss of the seminiferous tubules’ integrity, with a great amount of connective tissue between tubules (Figure 4C), with loss of germinal layers in the seminiferous epithelium, and a thickened basement membrane with an irregular profile. The interstitium was expanded by fibrosis and few inflammatory cells (Figure 4D).

#### 3.3.3. Behavioral Changes

**Figure 5:**
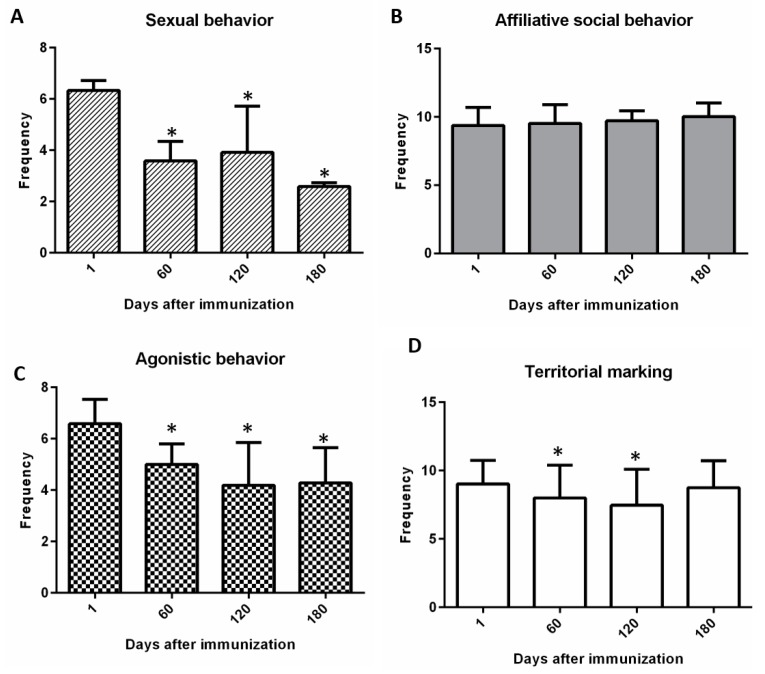
Vaccination changed the behavior of the vaccinated animals. Through surveys administered to owners on day 1, 60, 120, and 180 of the study, four behavioral categories were evaluated in the vaccinated dogs. Vaccination induced a decrease in sexual behaviors at days 60, 120, and 180 (**A**) but did not induce changes in affiliative behavior (**B**). Agonistic social behavior decreased during the entire study due to the vaccination (**C**) and territorial marking behavior decrease up to day 120 of the study (**D**). Asterisks (*) indicate significant differences in relation to day 0 (*p* < 0.05).

It was found—when evaluating changes in behavioral categories throughout the trial—that the sexual behaviors decreased from the second visit to the end of the trial (Figure 5A). Regarding social behavior evaluated, these did not show differences throughout the trial (Figure 5B). Conversely, the agonistic behaviors decreased compared to the first visit, from day 60 to day 180 (Figure 5C). Marking-associated behavior (Figure 5D) dropped up to day 120 and returned on day 180.

## 4. Discussion

For a long time, dog reproduction activity has been of great interest to human beings [31]. Canine immunocastration has been the object of research—among various reproductive control strategies—as a complementary method to surgical castration to control the population and, on the other hand, as a temporary and reversible reproductive and behavior control option [8,9,32].

Immunocastration induces antibodies production against the Gonadotropin-Releasing Hormone (GnRH), creating an immunological barrier between the hypothalamus and the adenohypophysis, thus blocking the normal action of the Gonadotropin-Releasing Hormone GnRH-I, the essential hormone in the reproductive system [33]. Before that, our laboratory corroborated that the GnRXG/Q antigen is immunogenic in rats, able to develop antibodies against GnRH-I when used together with chitosan polymer as an adjuvant [20,21,22]. This study confirms the immunogenic capacity of this molecule in dogs as target species, using chitosan as an adjuvant in two consecutive assays. In the first trial in controlled conditions, specific antibodies against the vaccine antigen were found which remained high until the end of the trial (day 270). However, when using the same formula in the second trial, with dogs with the owner in the field, the immunological reaction had a lower average duration of 150 days. The difference could be due to the less controlled aspects of the second trial, as the reaction to a vaccine can be influenced by a different individual and environmental factors such as genetics, age, stress, nutrition, and exposure to infectious disease, among others [34].

Earlier studies have shown that immunocastration causes changes in the immunized animals’ gonadal functionality [9,20,21,22,28,35]. In animals within controlled conditions, the evaluation of the vaccination effects on the testosterone serum levels revealed that this hormone dropped after vaccination and throughout the trial; like what other investigators found using other formulas for dog immunocastration [9,28].

In this sense, Donovan et al. obtained similar results, observing an increase of immunoglobulins against GnRH and a decrease in serum testosterone after vaccination in dogs with owners in a 20-week study [9]. Subsequently, Liu and colleagues in a study with controlled conditions evaluated a immunocastration formula for 28 weeks. These authors observed that the immunocastration induced an increase in GnRH antibodies, along with a decrease in testosterone and spermatogenesis [28]. Thus, we conclude that our results with controlled conditions are similar to those given in prior research in male dogs. 

However, in the field trial, the testosterone levels results varied between the participants and even in the same dog. This variability has already been documented by other researchers, reporting fluctuations of this hormone related to the different individual and external factors [36]. Future studies must define other testosterone complementary hormones such as the LH and FSH gonadotropins that are directly affected by immunocastration and their variations could provide more information regarding the immunocastrating effects of this vaccine [9,18,33].

On the other hand, gonadal function was evaluated with a spermiogram in controlled conditions, finding changes in the ejaculate of most of the assessed animals. However, when the gonadal function in the field trial was evaluated by histological gonadal characteristics only five of the 25 castrated dogs exhibited degenerative changes in the testicular parenchyma; this is probably because in most of the animals the immunocastrating effect was reverted by the circulating antibodies drop from day 180, i.e., a month before the surgical castration and histological analysis (day 210). It makes sense because the immunocastration effectiveness depends on the presence of enough antibodies to form immune complexes to prevent bonding of the GnRH-I hormone to its pituitary receptor, inhibiting the normal function of the hypothalamus-hypophysial-gonadal axis [33]. Therefore, it is necessary to continue improving the vaccination formula and to carry out new tests in field conditions, in search of a more potent immunocastration vaccine. A recommendable period would be at least 6–12 months of duration, so that this vaccination can be considered as a real alternative to surgical castration in dogs. Without a doubt, duration of the effect is one of the principal problems in applying this technology in field conditions. This is because in a previous study by Donovan and colleagues in dogs with owners, authors also showed a decrease in the immunocastrator effect at week 20 of the study [9].

Research regarding immunocastration effects analysis on behavior has revealed that there are not many, recording studies on pigs [13,37,38], bulls [16], stallions [39], and dromedaries [24], where the main evaluation was of sexual (libido, covering) and aggressive (threats, attacks) behavior; social behavior—important in gregarious and social species such as dogs—was not studied. Besides the variables related to sexual and marking deportment, this study addresses other conducts linked to the animal’s social behavior classified as associating and antagonistic behaviors that are considered as the most representative of a group’s correct behavior—together with sexuality and play. They are manifestations of cooperating or competitive forces, the cornerstone of their social order. This study is a pioneer in evaluating the immunocastration effect on dog behavior, although future studies should consider a higher number of behavioral groups. Additionally, an animal behavior specialist should make an additional visual evaluation of the animals, together with the questionnaires given to owners. 

## 5. Conclusions

This new canine immunocastrating vaccine proved to be safe, immunogenic, and able to reduce gonadal functionality, as well as sexual, marking, and agonistic behavior in the injected animals; emerging as a promising alternative to control reproduction and unwanted demeanor in dogs.

## Figures and Tables

**Table 1:** Spermiogram evaluation on day 270 of the trial.

Parameter	Dog 1	Dog 2	Dog 3	Dog 4	Dog 5	Dog 6	Dog 7	Control 1	Control 2
Forward motility	70 *	75	88	75	97	70 *	0 *	95	95
Sperm vitality	98	90	95	95	98	89	0 *	98	98
Normal morphology	>98	70 *	>95	>98	>98	60 *	0 *	95	95
Sperm concentration	135	50 *	49 *	110 *	350	72 *	0 *	149	152

(*) out of range values.
